# Comparison of ease of induction of spinal anaesthesia in sitting with legs parallel on the table versus traditional sitting position

**DOI:** 10.11604/pamj.2017.28.223.6992

**Published:** 2017-11-13

**Authors:** Jide Michael Afolayan, Peter Olufemi Areo, Patrick Temi Adegun, Kolawole Olubunmi Ogundipe, Aderemi Benjamin Filani

**Affiliations:** 1 Department of Anaesthesia, Ekiti State University Teaching Hospital, Ado-Ekiti, Nigeria; 2 Department of Surgery, Ekiti State University Teaching Hospital, Ado-Ekiti, Nigeria

**Keywords:** Spinal anaesthesia, elderly, spinal needle, bony contacts

## Abstract

**Introduction:**

It is sometimes difficult for some patients to optimally flex their hips and knees making traditional position for induction of spinal anaesthesia difficult to achieve. The ease of induction of spinal anaesthesia was compared with patients in sitting position with legs remaining on the table (new sitting method) versus legs placed on the side stool (traditional sitting method).

**Methods:**

One hundred eligible elderly patients, aged between 65 and 80 years, scheduled for open prostactectomy, were prospectively randomized to 2 groups, LS and LT. Patients in (LS group) had their spinal anaesthesia induced in sitting position with their legs placed on the stool while patients in (LT group) had their spinal anaesthesia induced in sitting position with their legs remaning on the operating table. The primary endpoint was correct needle placement. Numbers of attempts, needle redirections and patients' comfort were determined to compare outcome in the two groups.

**Results:**

More patients in LS group (78%) than those in the LT group (64%) had successful placement of spinal needle at first attempt (P = 0.12, RR = 1.6, 95% CI = 0.863-3.102). Needle redirections were similar at first attempt (52% versus 40%; P = 0.22). The groups were equivalent with respect to 100% overall success rate (P = 1.000). It took longer time to induce spinal anaesthesia in patients in LS group (240 vs 125s, p < 0.001). Patients in LT were more comfotable.

**Conclusion:**

The 100% overall success rate was comparable. However, patients were generally more comfortable with their legs placed on the table.

## Introduction

Generally, reduced lumbar lordosis during induction of spinal anaesthesia is a product of good positioning prior to the procedure [1]. Anecdotal evidence suggested that induction of spinal anaesthesia in a sitting position with legs placed on the table may produce better reversal of lumbar lordosis, decrease frequency of bony contacts and make spinal puncture easier [1,2]. Patients for spinal anaesthesia are traditionally positioned in a sitting posture on the operating table. A small stool is placed by the side of the operating table to support their legs. The forearms can be made to rest on the thighs with both hips and knees maximally flexed [3-5]. For some time now, Nigerian anaesthetists are fond of using non-traditional method of positioning in the placement of spinal needle. This non-traditional posture entails that the patients sit up from supine position without moving the two legs from the operating table, their hips maximally flexed and knees maximally extended [1, 2]. The vast increase in spinal anaesthesia for surgical procedure below the umbilicus and relative inability of some patients to flex knees has contributed to the frequent use of this non-traditional position as its proponents claim it saves time [6-8]. There are paucity of published research to compare the success of lumbar puncture associated with these postures, hence it is difficult to predict the outcome of each. Sitting, lateral and prone positions have been the traditional positions for spinal anaesthesia [8-12]. Several studies [4, 5, 9, 10] have compared traditional sitting position with lateral position for induction of spinal anaesthesia but none has investigated this non-traditional but modified sitting position by which patients sit on the operating table with their legs kept straight on the operating table despite its regular use by Nigerian anaesthetists.

## Methods

This prospective randomized study was carried out at the Ekiti State University Teaching Hospital, Ado-Ekiti, Nigeria. After institutional approval and informed written consent, one hundred elderly patients in the age group 65-80 years, belonging to the American Association of Anaesthesiologists (ASA) physical status score I or II, scheduled for elective prostatectomy, were included in the study. Patients with anatomical spinal deformity, inability to flex the knees, body mass index (BMI) > 30 kg/m2, neurological disease, coagulation disorders, local infection and unstable haemodynamics were excluded from the study. Preoperative assessment of the patients was carried out in the wards the day before surgery. This of a clinical history and physical examination. Mallampati and ASA Scores as well as the degree of flexion at knees and hip joints were determined. Laboratory investigations included a full blood count, serum electrolytes, urea and creatinine and urinalysis. In the pre-anaesthetic review on the day before surgery, patients were briefed about the proposed position for the procedure according to group allocation. All patients were fasted overnight and premedicated with oral diazepam 10mg a day before surgery. In the operating theatre, spinal pack was made available. Routine anaesthetic equipment check was carried out and anaesthetic machine with appropriate sizes of endotracheal tubes and laryngoscopes were made on stand-by. Baseline vital signs were recorded including systolic, diastolic, mean arterial blood pressures, oxygen saturation and continuous electrocardiographic monitoring, using electronic multiparameter monitor. Intravenous line was established for all the patients using a 18 gauge cannula and Ringers Lactate infusion was started. Spinal anaesthesia was performed either in the sitting position with the two legs on a stool beside the operating table or in the sitting position with the two legs maitained straight on the operating table. Patients were randomized into two groups of 50 patients each based on a computer-generated randomization system: in Group LT, patients were put in sitting position with the two legs placed straight on the operating table and in Group LS, patients were put in sitting position with legs placed on the side stool.

In the operating theatre, patients were preloaded with 12ml/kg Ringers Lactate and patients were positioned for subarachnoid block. Patients in Group LS were placed in a sitting position with the legs placed on a stool at the edge of the table with one pillow hugged around the chest. The patients in group LT were placed in sitting position with their two legs placed in a straight state on the operating table with one pillow hugged around the chest. The patients in the two groups were made to be in flex posture by bending the hip joints and flexing the neck towards knee as far as possible (forehead to knee position). An anaesthesia assistant helped each patient to obtain and maintain the best possible flexed posture by holding the patients at their occipital region and knees. Block was performed in any of the two positions. Patients in LS group sat on the table with their legs hanging from the edge of the table with the support of a stool under their feet. The height of the stool was adjusted to achieve flexion of the back.

All patients were asked to bend forward and arch out their back maximally. Arms were rested over a pillow which was kept on the lap. This prevented the patient from slumping to either side. Under aseptic conditions and following standard techniques of spinal anaesthesia, the second anaesthetist performed the blocks with a 25 G Quincke spinal needle. Using the iliac crest on both sides as the landmark, the anaesthetist had option of using any of the spaces for his first and the second preferred intervertebral space out of L2-L3, L3-L4 and L4-L5. In performing induction of spinal anaesthesia, the first attempt was achieved by introducing the spinal needle into first preferred interspinous space untill tactile sensation was felt. At this stage, no needle re-direction except for bony contacts. Correct placement of spinal needle into the subarachnoid space was judged by appearance of cerebrospinal fluid (CSF) in the hub of the needle. Appearance of free flow of CSF confirmed a successful needle insertion. In case there was either no CSF or there was scanty CSF, the following adjustments were carried out in order to achieve correct placement of the spinal needle. The needle would be rotated clockwise 90° and waited for 10s; another sequence of 900 rotation would be done if CSF was still not observed untill a total of four-quadrant rotations of 90° was achieved, amounting to 360° clockwise rotation, with an allowance of interval of 10s between each rotation. Inspite of these maneuvers, if there was no free flow of CSF, the anaesthetist would further advance the spinal needle untill another tactile sensation was felt. Also at this stage no needle re-direction except for bony contacts. A free flow of CSF confirmed correct placement otherwise the above outlined 3600 clockwise maneuver would be carried out. Inability to achieve free flow of CSF following second advancement, even after these maneuvers, would result in the needle being withrawn to a level just beneath the skin for re-direction cephalad, horizontally or caudally.

A maximum of three re-directions was allowed for each attempt. Following each re-direction and feeling of tactile sensation a free flow of CSF confirmed correct placement of spinal needle, otherwise, the above-mention 3600 maneuver was observed. Inability to observe flow of CSF at the hub of the spinal needle, despite three re-directions and three needle advancement coupled with 3600 maneuver, was followed by completely withdrawal of the spinal needle from the skin and this was considered as failed first attempt. Also whenever there were persistent bony contacts (more than three-times) despite cephalad, horizontal or caudal angulation of the needle, then the needle would be withdrawn completely from the skin. This was also termed failed first attempt.

The number of times for needle re-directions and bony contacts was documented. Following completely removal of the needle after failed first attempt, a reassessment of the midline and the second most preferred interspinous space was located. Second attempt was carried out by re-introducing the spinal needle to the second most preferred intervertebral space untill another tactile sensation was felt. Its correct placement was confirmed by free flow of CSF otherwise the second advancement of needle or needle re-directions coupled with 3600 maneuvers were carried out step-by-step as outlined for first attempt.

Following the above-mentioned maneuvers, if there was no free flow of CSF then the needle would be withddrawn completely from the skin and this was considered as failed second attempt. Also whenever there were persistent bony contacts (more than three-times) despite cephalad or caudal angulation of the needle, then the needle would be withdrawn completely from the skin. This was also termed failed second attempt. The number of times for needle re-directions and bony contacts was documented. Following failed second attempt, the third and the final attempt was considered in first or second preferred space. Another reassessement was carried out in order to facilitate correct placement. In order to establish third attempt at spinal anaesthesia, the spinal needle was advanced into either of the first or second most preferred interspinous space to look out for free flow of CSF at the hub of the needle. A free flow of CSF confirmed correct placement. Lack of free flow of CSF prompted the anaesthetist to carry out the second advancement of needle or needle re-directions coupled with 3600 maneuvers step-by-step as outlined for first attempt.

Following the above-mentioned maneuvers, second advancement and needle re-direction coupled with 3600 maneuver of the needle, if there was no free flow of CSF then the needle would be withdrawn completely from the skin and this was considered as failed third and final attempt. Also whenever there were persistent bony contacts (more than three-times) despite cephalad or caudal angulation of the needle then the needle would be withdrawn completely from the skin. This was also termed failed third attempt. The number of times for needle re-directions and bony contacts was documented. Thus, considering all the above-mentioned manipulations, each attempt was considered as a failed attempt if: (i) there was no CSF in the hub, despite advancement, three re-directions coupled with 3600 maneuver of the needle (ii) when the needle could not be negotiated because of three repeated bony contacts. Following intrathecal administration of 3ml of hyperbaric bupivacaine, patients were returned into supine position. Maximum sensory block height was assessed at every two minutes untill T6 was achieved, using loss of sensation to cold and gentle pin prick test. A minimum sensory block height of T6 was the minimum desired level for commencement of surgery. Also documented was time to induce spinal which was the time from completion of preload until patient was returned into supine position.

Overall onset time which was the time from when the spinal needle was introduced until T6 analgesia was achieved. The following parameters were recorded: overall success, attempts required, number of patients requiring manipulation of needle and the type of manipulations. Complications such as hypotension, headache and backache were noted by the anaesthetist during and after the operation. Primary outcome measure was the number of attempts during correct needle placement. Each attempt was considered as a failed attempt if: (i) there was no CSF in the hub, despite advancement, three needle re-directions coupled with 3600 maneuver of the needle (ii) when the needle failed to advance because of three repeated bony contacts. Additional data including onset of sensory block, side effects and patients' comfortability was assessed (using a 10cm Visual Analogue Score) and documented. 10cm denoted maximal comfort while 0cm denoted minimal comfort. These primary and secondary outcomes were compared in the two groups.

Statistical analysis: Normally distributed numerical variables among the two groups were analyzed with unpaired Student's t-test. Proportions and median were analyzed respectively with Chi-square or Fisher's exact tests and Mann-Whitney test respectively. In all calculations, P < 0.05 is the level of significance. The primary objective of the study was to decrease the incidence of bony contacts from 50% ( as reported by Fisher et al [1]) while using traditional sitting position to 7% if patient was in sitting position with his two lower limbs placed on the operating table. On the basis of this, a power analysis indicated that a minimum of 50 subjects per group would be sufficient to detect this difference in incidence of bony contacts with a study power of 80% and α = 0.05

## Results

As shown in Table 1, age, BMI and ASA physical status were comparable among the patients in the two groups. According to Table 2, onset of analgesia was faster in patients whose legs were placed on the table than in patients whose legs were placed on the side stool. It significantly took a longer time to institute spinal block in patients whose legs were placed on the side stool. However, patients in LT group significantly consumed more ephedrine. Intraoperative clinical variables were shown in Table 3. The proportion of patients with needle redirections (52% versus 40%; P = 0.22, RR = 1.3, 95% CI = 0.844-2.000) and bony contacts (34% versus 22%; P = 0.18, RR = 1.5, 95% CI = 0.807-2.958) at first attempt among the two groups were comparable respectively. More patients in LS group (78%) than those in the LT group (64%) had successful placement of spinal needle at first attempt (P = 0.12, RR = 1.6, 95% CI = 0.863-3.102). A significant proportion of patients in LT group (24%) compared to LS group (08%) had needle re-directions at second attempt (P = 0.03, RR = 3, 95% CI = 1.038-8.673). Needle redirections, bony contacts and successful placement are comparable among the patients in the two groups. For the two positions, the overall success rate for the placement of spinal neddle was 100% (P = 1.000). Most patients in LT group (44%) are significantly more comfortable with their legs maintained on the table than those in LS group (33%) whose legs were placed on the side stool (P = 0.01, RR = 0.4, 95% CI = 0.152-.0.821). As shown in Table 4, the incidence of hypotension, bradycardia, shivering were comparable among the patients in the two groups.

**Table 1:** Patients’ characteristics

	**LT**	**LS**	**P value**
**Age (years) (mean SD)**	69 (± 7)	65 (±10)	0.76
**Body Mass Index (BMI) (kg/m^2^ )**	26 (± 3.8)	25 (± 4.5)	0.42
**American Society of Anesthesiologists (n/%)**			
1	31 (62)	22 (44)	
11	19 (38)	28 (56)	0.07

**Table 2:** Block characteristics of the patients

	LT (median and range	LS (median and range)	P value
**Analgesic block at T_6_(min)**	3 (2, 6)	6 (3, 8)	< 0.001
**Time to induce spinal (sec)**	125 (90, 140)	240 (180, 390)	< 0.001
**Overall onset time (min)**	10 (8, 12)	12 (10, 14)	< 0.001
**Total ephedrine required (mg)^x^**	12 (9,15)	6 (3, 12)	< 0.001

**Time to induce spinal** = time from completion of preload untill patient was completely returned to supine position. **X** = in the first 15 min following injection. **Overall onset time** = time from advancement of needle until spinal block of T_6_ was achieved.

**Table 3:** Intraoperative clinical variables

	**LT (n/%)**	**LS (n/%)**	**RR**	**95% CI**	**P value**
**First attempt patients with**					
Needle re-directions	26 (52)	20 (40)	1.3	0.844-2.000	0.22
Bony contacts	17 (34)	11 (22)	1.5	0.807-2.958	0.18
Successful placement	32 (64)	39 (78)	1.6	0.863-3.102	0.12
**Second attempt patients with**					
Needle re-directions	12(24)	4 (08)	3.0	1.038-8.673	0.03
Bony contacts	7(14)	4 (08)	1.8	0.512-6.848	0.34
Successful placement	16(32)	11 (22)	0.9	0.686-1.109	0.26
**Third attempts patients with**					
Needle re-directions	2 (4)	0 (0)	5.0	0.246-101.590	0.29
Bony contacts	0 (0)	0 (0)	1.0	0.020-49.438	1.00
Successful placement	2 (4)	0 (0)	5.0	0.246-101.590	0.29
Overall success	50 (100)	50 (100)	1.0	0.020-49.438	1.00
**Comfortability (10cmVAS)**					
Greater or equal 5	44(88)	33(66)	0.4	0.152-0.821	0.01
Less than 5	6(12)	17(34)			

**RR** = Relative Risk, **CI** = Confidence Interval, **VSA** = Visual Analogue Score

**Table 4:** Intraoperative complications

	LT	LS	P value
Hypotension	31	38	0.13
Bradycardia	0	4	0.14
Shivering	2	1	0.57
High spinal	0	0	1.00

## Discussion

According to this present study, correct placement of spinal needle is possible in the elderly in sitting position with the two legs placed on the operating table. Although, more patients in group with legs placed on the stool (78%) than those in whose legs were placed on the table (64%) had successful placement of spinal needle at first attempt; the overall success rate was hundred percent and comparable among the two groups. Our study also demonstrated that onset of analgesia was faster in patients whose legs were placed on the table than in patients whose legs were placed on the side stool. It significantly took a longer time to institute spinal block in patients whose legs were placed on the side stool. However, patients in whose legs were placed on the table significantly consumed more ephedrine in the first 15 minutes following injection of bupivacaine. Most anaesthetists in our centre preferred to place the legs of their patients on the table because they believe that it reduces time to place spinal anesthesia especially in fetal distress [7], allows full length of legs on the table which helps to stabilize unstable patient such as drowsy eclamptic for spinal anesthesia [6], reduces time wasting in searching for side stool and turning of patient to the side of the operating table. All these turnings of patients may constitute unnecessary stress on anaesthetist. The time that required to search for stool, move the patient to the side and arrange the foot on the stool is eliminated in the hamstring stretch position [2, 13-16]. As entertained by its proponents, this present study corroborated the fact that time to induction of spinal anaesthesia was shorter in patients whose legs were placed on the table than in patients whose legs were put on side stool. The time taken to move the legs to the side stool and to return them to the operating tables among other turnings might be responsible for the difference in the time for induction. Incidence of spinal needle bony contacts is more in the elderly than average middle age population [17-20]. Correct identification of spinal space, reduction in the number of spinal needle-bone contacts and needle re directions are possible with appropriate reduction in lumbar lordosis during induction of regional block in the elderly [1, 13, 14, 17, 20-22]. Tashayod and co-workers [2], compared correct placement of spinal needle with patients in the sitting position with legs placed on the table and the traditional sitting position of putting the foot on the side stool. They found that the modified sitting position of placing the legs on the table with maximum extension of knees, adduction of hips, and forward bending (hamstring stretch position) was more effective in reducing lordosis of lumbar spine and making spinal puncture easier [2].

Researchers observed that putting the legs on the table helped in the reversal of lumbar lordosis by increasing hamstring tension, tilting the pelvis, and reducing lumbar curvatures [2,13]. In contrast to our study and that of Fisher et al [1], Tashayod et al [2] found that ease of placement of spinal needle was better in hamstring stretch position ( patients' legs on the table) than in traditional position (patients' legs placed on the side stool). Our study showed that correct placement of spinal needle is possible in the elderly while the legs were placed on the operating table and the result was comparable between the two groups. Although, more patients in group with legs placed on the stool (78%) than those in whose legs were placed on the table (64%) had successful placement of spinal needle at first attempt, the overall sucess was equivalent. This difference was probably because their patients were midle-aged and could lean forward maximally. The osteo-arthritic changes in the elderly may prevent correct flexion or extension of knee joints as well as prevention of maximal forward bending resulting in less hip flexion and reversal of lordosis. Fisher et al [1] also affirmed that the number of needle bony contact was lower in the patients whose lower limbs were placed on the table. In our study, more people clinically but not statistically significant had bony contacts in the group with legs on the table. This difference might be probably because larger epidural needles were used in the obstetric population studied by Fisher et al1 compared to smaller spinal needles used in elderly population in our study. Bony contacts are more in elderly compared to midle-aged adult population [1, 17, 18, 20]. However, patients in whose legs were placed on the table significantly consumed more ephedrine in the first 15 minutes following injection of bupivacaine [4, 6-8]. It has been that incidence of hypotension is more in the first 15 minutes of injection of heavy bupivacaine. Shorter onset of block coupled with rapid cephalad movement of block might be responsible for increased consumption of ephedrine among the patients whose legs were placed on the operating table. Elderly patients in modified sitting position were significantly more comfortable with the position for spinal anaesthesia than those in the traditional position. The patients in hamstring stretch were significantly more comfortable because hip flexion and forward lean were limited by osteo-arthritic changes in the elderly making flexion of the knee incomplete. This study was able to justify that the modified sitting position, where patients put legs on the table prior to induction, saved time to induction of spinal anaesthesia as claimed by its proponents. However the study was limited because of lack of blinding. Although correct placement of spinal needle is possible while patient is sitting with the two legs placed on the table, further study is required in obstetric population because of the probable limited flexion of the hip due to gravid uterus.

## Conclusion

Correct placement of spinal needle is possible in the elderly in sitting position with the two legs placed on the operating table. Although, more patients in group with legs placed on the stool than those in whose legs were placed on the table had successful placement of spinal needle at first attempt; the overall success rate of hundred percent was comparable among the two groups.

### What is known about this topic

- Traditional method of putting the two legs on side stool during induction of spinal anaesthesia is excellent;
- There is high succes rate following this position.

### What this study adds

- Induction of spinal anaesthesia, with patient's legs placed on theatre table, also yields excellent result;
- High success rate in this new position is also observed, however, patients are more comfortable with their two legs placed on the theatre tables;
- Time to establish spinal anaesthesia is faster in this new position.

## Competing interests

The authors declare no competing interests.

## References

[1] Fisher KS, Arnholt AT, Douglas ME, Vandiver SL (2009). A randomized trial of the traditional sitting position versus the hamstring stretch position for labor epidural needle placement. Anesth Analg..

[2] Tashayod ME, Tamadon S (1980). Spinal block in sitting position without moving the legs. Middle East J Anesthesiol..

[3] Mohammad SS, Hassan M, Marashi SM (2014). Comparing the squatting position and traditional position for ease of spinal needle placement: a randomized clinical trial. Anesth Pain Med..

[4] Obasuyi B, Fyne-face S, Mato CN (2013). A comparison of the haemodynamic effects of lateral and sitting positions during induction of spinal anaesthesia for caesarean section. Int J Obstet Anesth..

[5] Shahzal K, Afshan G (2013). Induction position for spinal anaesthesia: sitting versus lateral position. J Pak Med Assc..

[6] Afolayan Jm, Nwachukwu CE, Esangbedo ES, Omu OP, Amadasun FE, Fadare JO (2014). Evolving pattern of spinal anaesthesia in stable eclamptic patients undergoing caesarean section at University of Benin Teaching Hospital, Benin, Nigeria. Niger J Med..

[7] Afolayan JM, Olajumoke TO, Esangbedo SE, Edomwonyi NP (2014). Spinal anaesthesia for caesarean section in pregnant women with fetal distress: time for reappraisal. Inter J Biomed Sci..

[8] Biswas BK, Agarwal B, Bhattarai B, Dey S, Bhattacharyya P (2012). Straight versus flex back: does it matter in spinal anaesthesia?. Indian J Anaesth..

[9] Russell IF (1996). Spinal anaesthesia: sitting or lateral positions. Anaesth..

[10] Fernandez R, Taboada M, Ulloa B, Rodriquez J, Masid A, Alvarez J (2010). Needle-induced paresthesia during single spinal anaesthesia: a comparison of sitting versus lateral decubitus position. Reg Anesth Pain Med..

[11] Laakso E, Pitkanen M, Kytta J, Rosenberg PH (1997). Knee-chest versus horizontal side position during induction of spinal anaesthesia in patients undergoing disc surgery. Br J Anaesth..

[12] Badolov P, Celebi H, Mentes B (2005). Comparison of spinal anaesthesia with isobaric bupivacaine in the prone or jacknife position with hyperbaric 0,5% bupivacaine in sitting position for anorectal surgery. Gazi Med J..

[13] Stokes IAF, Abery JM (1980). Influence of hamstring muscles on lumbar spine curvature in sitting. Spine..

[14] DeOliveira GR, Gomes HP, da Fonseca MH, Hoffman JC, Pederneiras SG, Garcia JH (2002). Predictors of successful neuroaxial block; a prospective study. Eur J Anaesthesiol..

[15] Sandoval M, Shestak W, Sturmann K, Hsu C (2004). Optimal patient position for lumbar puncture, measured by ultrasonography. Emerg Radiol..

[16] Platt F, Hall M (2001). Hip flexion for lumbar puncture-not easy for obstetric practice. Anesthesia..

[17] Fredman B, Zohar E, Rislick U, Sheffer O, Jedeikin R (2001). Intrathecal anaesthesia for the elderly: influence of induction position on perioperative haemodynamic stability and patient comfort. Anaesth Intensive Care..

[18] Baigmohammadi MT, Khan ZH (2007). Modified sitting position: a new position for spinal anesthesia. Anesth Analg..

[19] Schelew BL, Vaghadia H (1996). Ankylosing Spondylitis and neuroaxial anesthesia: a 10 year review. Can J Anaesth..

[20] Fisher A, Lupu L, Gurevitz B, Brill E, Margolin E, Hertzanu Y (2001). Hip flexion and lumbar puncture: a radiological study. Anaesthesia..

[21] Miro M, Guasch E, Gilsanz F (2008). Comparison of epidural analgesia for labor: a retrospective study of 6497 cases. Inter J Obstet Anesth..

[22] Tarkkila P, Huhtala J, Salminen U (1994). Difficulties in spinal needle use: insertion characteristics and failure rates associated with 25-, 27- and 29-gauge Quincke-type spinal needles. Anaesthesia..

